# Whole Genome DNA Sequence Analysis of *Salmonella* subspecies *enterica* serotype Tennessee obtained from related peanut butter foodborne outbreaks.

**DOI:** 10.1371/journal.pone.0146929

**Published:** 2016-06-03

**Authors:** Mark R. Wilson, Eric Brown, Chris Keys, Errol Strain, Yan Luo, Tim Muruvanda, Christopher Grim, Junia Jean-Gilles Beaubrun, Karen Jarvis, Laura Ewing, Gopal Gopinath, Darcy Hanes, Marc W. Allard, Steven Musser

**Affiliations:** 1 Center for Food Science and Applied Nutrition (CFSAN), Food and Drug Administration (FDA), College Park, Maryland, United States of America; 2 Battelle Memorial Institute, Crystal City, Virginia, United States of America; 3 Division of Virulence Assessment (DVA), Virulence Mechanisms Branch (VMB), CFSAN/FDA, Office of Applied Research and Safety Assessment (OARSA), Laurel, Maryland, United States of America; Cornell University, UNITED STATES

## Abstract

Establishing an association between possible food sources and clinical isolates requires discriminating the suspected pathogen from an environmental background, and distinguishing it from other closely-related foodborne pathogens. We used whole genome sequencing (WGS) to *Salmonella* subspecies *enterica* serotype Tennessee (*S*. Tennessee) to describe genomic diversity across the serovar as well as among and within outbreak clades of strains associated with contaminated peanut butter. We analyzed 71 isolates of *S*. Tennessee from disparate food, environmental, and clinical sources and 2 other closely-related *Salmonella* serovars as outgroups (*S*. Kentucky and *S*. Cubana), which were also shot-gun sequenced. A whole genome single nucleotide polymorphism (SNP) analysis was performed using a maximum likelihood approach to infer phylogenetic relationships. Several monophyletic lineages of *S*. Tennessee with limited SNP variability were identified that recapitulated several food contamination events. *S*. Tennessee clades were separated from outgroup salmonellae by more than sixteen thousand SNPs. Intra-serovar diversity of *S*. Tennessee was small compared to the chosen outgroups (1,153 SNPs), suggesting recent divergence of some *S*. Tennessee clades. Analysis of all 1,153 SNPs structuring an S. Tennessee peanut butter outbreak cluster revealed that isolates from several food, plant, and clinical isolates were very closely related, as they had only a few SNP differences between them. SNP-based cluster analyses linked specific food sources to several clinical S. Tennessee strains isolated in separate contamination events. Environmental and clinical isolates had very similar whole genome sequences; no markers were found that could be used to discriminate between these sources. Finally, we identified SNPs within variable *S*. Tennessee genes that may be useful markers for the development of rapid surveillance and typing methods, potentially aiding in traceback efforts during future outbreaks. Using WGS can delimit contamination sources for foodborne illnesses across multiple outbreaks and reveal otherwise undetected DNA sequence differences essential to the tracing of bacterial pathogens as they emerge.

## Introduction

Salmonella enterica one of the most common causes of foodborne illness outbreaks. Although most serotypes are able to cause human disease, only about 20 of the over 2,500 identified Salmonella serotypes are typically associated with human disease. [1,2,3]. However, even serotypes that are infrequently reported can become significant threats to public health. For example, The Tennessee serovar has historically been uncommon among the *Salmonella* serotypes reported from food sources. In fact, the average reported cases of S. *enterica* Tennessee infection once represented only about 0.01% of all reported *Salmonella* serotypes [2]. Between 1994–2004, there were only 52 cases in which *S*. Tennessee was the main cause of foodborne infections [2], and only one outbreak of *S*. Tennessee infection, associated with powdered milk products and infant formula was reported to the Centers for Disease Control (CDC) in 1993 [4,5].

However, in November 2006, public health officials at CDC and state health departments detected a substantial increase in the reported incidence of isolates of *Salmonella* serotype Tennessee. As of May 22, 2007, a total of 628 persons infected with an outbreak strain of *Salmonella* serotype Tennessee had been reported from 47 states since August 1, 2006. In a multistate case-control study conducted during February 5–13, 2007, illness was strongly associated with consumption of either of two brands (Brand 1 and Brand 2) of peanut butter produced at the same plant [2,4,6,7]. Based on these findings, the plant ceased production and recalled both products on February 14, 2007 [6,8,9]. The outbreak strain of *Salmonella* Tennessee was subsequently isolated from several opened and unopened jars of Brand 1 and Brand 2 peanut butter and from environmental samples obtained from the plant. New case reports decreased drastically after the product recall.

In 2008–2009, a second national outbreak associated with peanut butter occurred. In these cases the peanut butter was found to have been contaminated with *Salmonella* Typhimurium. Larger numbers of children were infected in these later cases [6,10]. Interviews conducted with infected patients revealed that the outbreak occurred within 3 large institutions (2 care facilities and 1 elementary school) where the patients ate their meals [6]. Further investigation and review of food menus revealed a common food source eaten by infected patients [6]. Interestingly, during this outbreak investigation, CDC’s PulseNet identified and confirmed the presence of *Salmonella* serotypes other than Typhimurium in both food and environmental samples. Further investigation determined that an *S*. Tennessee isolate detected during this second outbreak had a pulse-field gel electrophoresis (PFGE) pattern that was indistinguishable from those S. Tennessee outbreak strains found during the 2006–2007 outbreaks, obtained from unopened and opened jars of one of the same brands of peanut butter. These findings suggested a possible association between the two outbreaks, despite being separated by an approximately two-year time frame [6,10]. Interestingly, the two implicated production plants are located approximately 70 km from one another. However, in the later outbreak the *S*. Tennessee strains were not directly associated with human illness [6,10].

If one accepts a common-source hypothesis of the *S*. Tennessee serovars in these outbreaks, it demonstrates not only the potential for widespread illness arising from locally contaminated products which are then broadly distributed, but also the possibility of illnesses arising from bacterial serovars that have not been previously implicated in major foodborne illness outbreaks in the United States. From what is known about the ability of *Salmonella* to thrive in particular environments, this hypothesis is reasonable. These organisms may contaminate peanuts during growth, harvest, or storage, and are able to survive high temperatures in a high-fat, low-water environment [11]. Therefore, although peanut butter typically undergoes heat treatment up to temperatures >158°F (>70°C), such heating may not always eliminate salmonellae [12]. It is also possible that processed peanut butter may be contaminated by bacteria that enter the production environment after heat treatment is complete, through raw peanuts or other sources, such as animals in the production plant. The bacteria may be brought into the plant on containers, humans from the outside environment, or other ingredients used to make peanut butter. These outbreaks suggest that the contamination of processed foods can occur after a heat-treatment step, underscoring the need for additional preventive controls in food-processing plants, and ongoing food safety surveillance.

Establishing an association between possible sources of food contamination and clinical isolates requires discriminating the suspected pathogen from the environmental background, and distinguishing it from other closely-related foodborne pathogens [13–16, 17–21]. The accurate subtyping and subsequent clustering of bacterial isolates associated with a foodborne outbreak event is important for a successful epidemiological investigation and the eventual traceback to a specific food or environmental source. However, phylogenetically closely related strains from a phylogenetic perspective can confound these investigations because of the limited genetic differentiation among serovars, such as *Salmonella* Enteritidis [22–29, 30]. Therefore, to provide a more rigorous analysis of the diversity found within these outbreaks, we performed the first whole genome DNA sequence analysis of *S*. Tennessee outbreak strains, and proceeded to perform a detailed phylogenetic analysis.

We performed whole genome shotgun sequencing (WGS) on isolates related to the *S*. Tennessee-peanut butter outbreak and other isolates derived from the same serovar. Samples of *S*. Tennessee obtained from cilantro food sources were sequenced for comparative purposes. Whole genome shotgun sequencing is an emerging molecular epidemiological tool [30–34]. Recent studies have shown that the voluminous amount of DNA sequence data accumulated via WGS can be used to distinguish among very closely related isolates, far beyond what close inspection of PFGE patterns and MLVA typing can reveal [30]. Further, WGS can identify the nature of the specific molecular difference(s) among sets of isolates, leading to the identification of characteristics that can be placed onto phylogenetic trees to show evolutionary relationships among the taxa under scrutiny. The phylogenetic trees can also serve the purpose of showing, in graphical form, the scale of the evolutionary distances between isolates that have different PFGE patterns.

**Fig 1:**
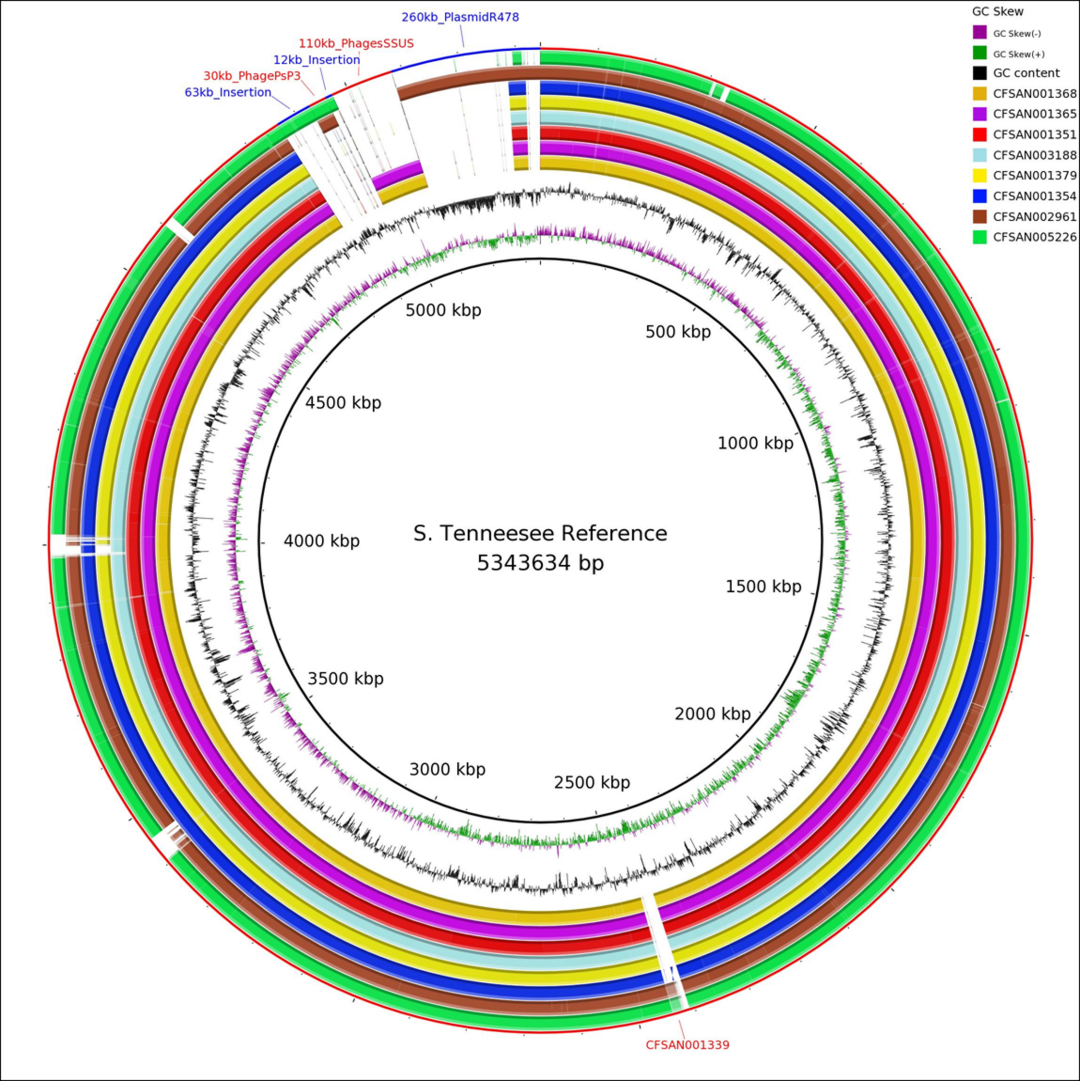
Whole genome alignment showing placement of mobile elements (outer rings) in representative samples of this study, GC skew (inner ring) and GC content by strand (second ring).

**Fig 2:**
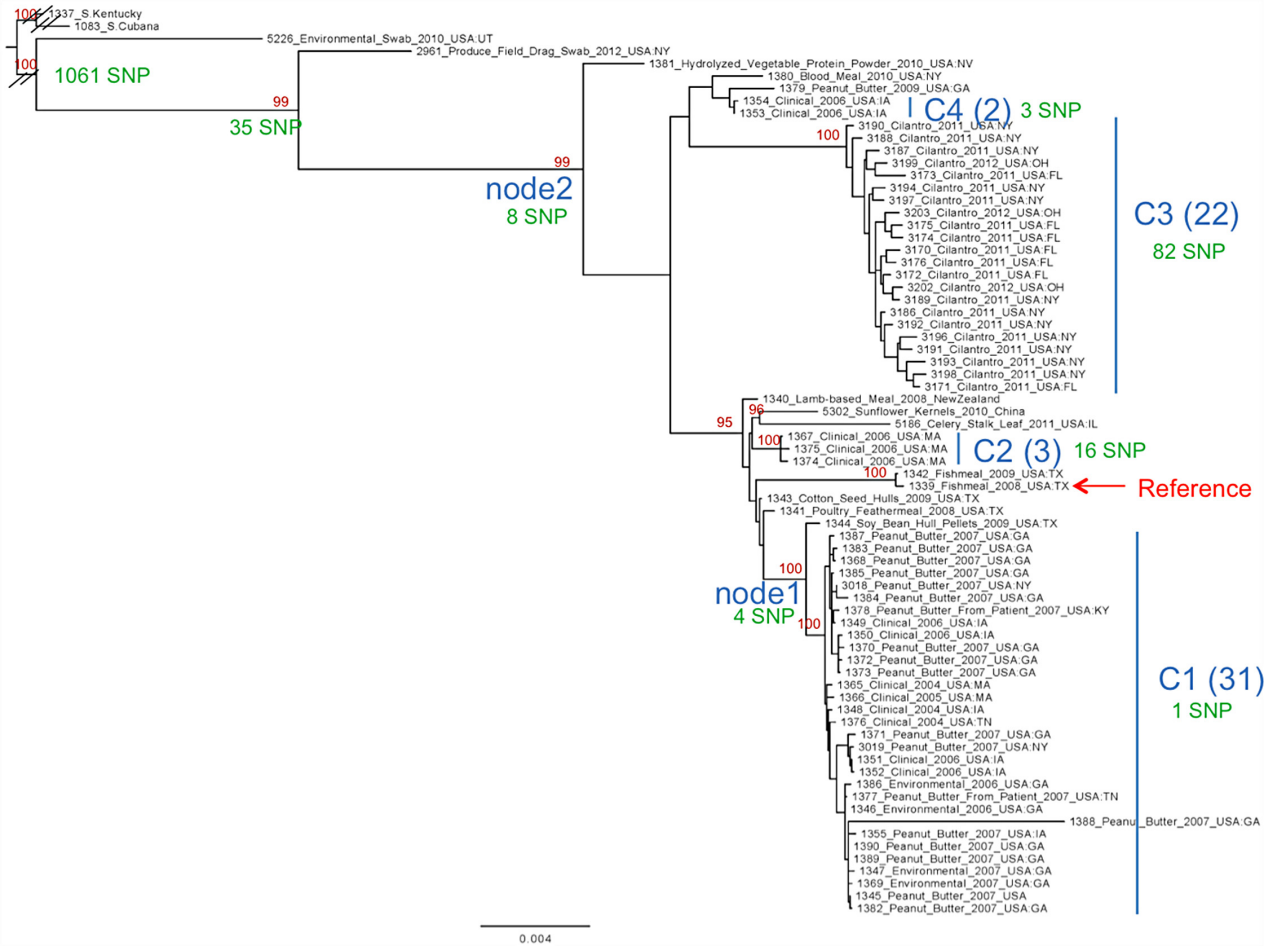
Cladogram of *S*. Tennessee serovar diversity showing major clades C1-C4, and the number of SNPs (in green) defining and residing within each clade.

In order to evaluate how WGS could assist in the identification of these isolates, we generated one closed genome sequence and 70 draft genomes of S. Tennessee isolates, including 28 isolates with two different PFGE patterns (JNXX01.0011 and JNXX01.0010) from the peanut butter outbreak, four related historical clinical isolates, eight environmental isolates with matching PFGE JNXX01.0011 profiles, three internal isolates, and 28 background isolates to establish the phylogenetic context of the diversity. Fig 1 shows the genome organization while Fig 2 depicts the phylogenetic results from these analyses.

## Materials and Methods

### Growth of bacterial strains, and genomic and plasmid DNA isolation

Genomic DNA was isolated from overnight cultures as follows: each initial pure culture sample was taken from frozen stock, plated on Trypticase Soy Agar, and incubated overnight at 37^°^C. After incubation, cells were taken from the plate and inoculated into Trypticase Soy Broth and cultured for DNA extraction. All samples were representative cultures from a full-plate inoculation and were not single colonies. Genomic DNA was extracted using Qiagen DNeasy kits.

The cilantro samples were provided through the U.S. Department of Agriculture (USDA) Microbiological Data Program (MDP). Samples collected in Michigan, Florida, New York, Ohio, and Washington were shipped overnight at room temperature and processed immediately upon receipt for the presence of *S*. *enterica*.

Cilantro was weighed into sterile Whirl-Pak bags, 100 g per sample, and 500 ml of modified Buffered Peptone Water (mBPW) [35] was added to each bag. The samples were manually mixed for 2 min and then incubated overnight at 37°C. The overnight enrichment cultures were subcultured into Tetrathionate Broth (TB) and Rappaport-Vassiliadis (RV) media and incubated according to the Bacteriological Analytical Manual (BAM) Chapter 5 *Salmonella* [36]. Following overnight incubation the TB and RV cultures were streaked onto Hektoen Enteric (HE), Xylose-Lysine-Tergitol 4 (XLT-4), and Bismuth Sulfite (BS) agar plates and the plates were incubated overnight at 37°C. Colonies demonstrating typical *S*. *enterica* morphology on each selective agar plate were subcultured onto 5% Sheep Blood Agar (SBA) plates for further characterization.

Colonies from SBA plates were confirmed as *Salmonella* using the Vitek® 2 Compact. The serotype was determined using the Premitest ® following the manufacturer’s instructions and a PCR serotyping method [37]. The PFGE pattern for each isolate was also determined using the CDC method for *S*. *enterica*.

### Library construction and genome sequencing

For this study, 71 *S*. Tennessee isolates from a variety of sources were sequenced. Of these, 42 isolates were shotgun sequenced using the Roche 454 GS Titanium NGS technology [38]. Each isolate was run on one quarter of a Titanium plate, producing roughly 250,000 reads per draft genome and providing an average genome coverage of ~20X. Illumina MiSeq^TM^ was used to sequence 28 isolates. The remaining isolate served as our reference for mapping; it was used to prepare a single 10 kb library following the Pacific Biosciences sample preparation methods for C2 chemistry. That 10 kb library was then sequenced using PacBio RS II on 4 single-molecule real-time (SMRT) cells using a 120-minute collection protocol, which provided a closed genome with an average genome coverage of > 200X. Our taxon sampling also included one *S*. Kentucky and one S. Cubana genome (Table 1), which were sequenced using Roche 454 GS Titanium and Illumina Miseq^TM^ chemistries, respectively. These two *Salmonella* serotypes, Cubana (Genbank accession APAG0000000) and Kentucky (Genbank accession AOYZ00000000) had previously been shown to be close relatives to *S*. Tennessee [39], and hence served as outgroups in this study.

**Table 1:** Isolates and metadata from the peanut butter-derived sources, including samples obtained from outbreak-associated foods and clinical samples.

Metadata associated with isolates in this study							Accession no(s)		
Tree Label	Salmonella enterica subsp. enterica Serovar and Strain	Collect-ion location	Isolation source	PFGE pattern (primary enzyme)	PFGE pattern (secondary enzyme)	Plat_ form	Bio-Project	WGS	SRA
1339_Fishmeal_2008_USA:TX(PB)	Tennessee str. CFSAN001339	USA:TX	fishmeal	JNXX01.0011		Pacbio	65249	AOXR00000000	SRR955294
1340_Lamb-based_Meal_2008_NewZealand(PB)	Tennessee str. CFSAN001340	New Zealand	meat and bonemeal, lamb-based	JNXX01.0011		454	65247	AOXQ00000000	SRR955295
1341_Poultry_Feathermeal_2008_USA:TX(PB)	Tennessee str. CFSAN001341	USA:TX	poultry feathermeal, hydrolized	JNXX01.0011		454	183850		SRR1048310
1342_Fishmeal_2009_USA:TX(PB)	Tennessee str. CFSAN001342	USA:TX	fishmeal	JNXX01.0011		454	183848		SRR1048336
1343_Cotton_Seed_Hulls_2009_USA:TX(PB)	Tennessee str. CFSAN001343	USA:TX	cotton seed hulls	JNXX01.0011		454	183851		SRR1048291
1344_Soy_Bean_Hull_Pellets_2009_USA:TX(PB)	Tennessee str. CFSAN001344	USA:TX	soy bean hull pellets	JNXX01.0011		454	183847		SRR1048289
1345_Peanut_Butter_2007_USA	Tennessee str. CFSAN001345	USA	peanut butter			454	66695		
1346_Environmental_2006_USA:GA(PB)	Tennessee str. CFSAN001346	USA:GA	environmental	JNXX01.0011		454	183850		
1347_Environmental_2007_USA:GA(PB)	Tennessee str. CFSAN001347	USA:GA	environmental	JNXX01.0011		454	183848		
1348_Clinical_2004_USA:IA(PB)	Tennessee str. CFSAN001348	USA:IA	stool	JNXX01.0011		454	183851		SRR1048318
1349_Clinical_2006_USA:IA(PB)	Tennessee str. CFSAN001349	USA:IA	stool	JNXX01.0011	JNXA26.0001	454	183847		SRR1048241
1350_Clinical_2006_USA:IA(PB)	Tennessee str. CFSAN001350	USA:IA	stool	JNXX01.0011	JNXA26.0001	454	183850		SRR1048273
1351_Clinical_2006_USA:IA(PB)	Tennessee str. CFSAN001351	USA:IA	stool	JNXX01.0011		454	183848		SRR1048258
1352_Clinical_2006_USA:IA(PB)	Tennessee str. CFSAN001352	USA:IA	missing	JNXX01.0011	JNXA26.0001	454	183851		
1353_Clinical_2006_USA:IA(PB)	Tennessee str. CFSAN001353	USA:IA	urine sample	JNXX01.0011	JNXA26.0001	454	183847		SRR1048254
1354_Clinical_2006_USA:IA(PB)	Tennessee str. CFSAN001354	USA:IA	stool	JNXX01.0011	JNXA26.0001	454	183850		SRR1048250
1355_Peanut_Butter_2007_USA:IA(PB)	Tennessee str. CFSAN001355	USA:IA	peanut butter	JNXX01.0011		454	183848		SRR1048333
1365_Clinical_2004_USA:MA(PB)	Tennessee str. CFSAN001365	USA:MA	stool sample	JNXX01.0010		454	183851		SRR1048252
1366_Clinical_2005_USA:MA(PB)	Tennessee str. CFSAN001366	USA:MA	stool sample	JNXX01.0026		454	183847		SRR1048329
1367_Clinical_2006_USA:MA(PB)	Tennessee str. CFSAN001367	USA:MA	wound	JNXX01.0011	JNXA26.0001	454	183850		SRR1048246
1368_Peanut_Butter_2007_USA:GA(PB)	Tennessee str. CFSAN001368	USA:GA	peanut butter	JNXX01.0010		454	183848		SRR1048300
1369_Environmental_2007_USA:GA(PB)	Tennessee str. CFSAN001369	USA:GA	environmental	JNXX01.0011		454	183851		SRR1048323
1370_Peanut_Butter_2007_USA:GA(PB)	Tennessee str. CFSAN001370	USA:GA	peanut butter	JNXX01.0011		454	183847		SRR1048330
1371_Peanut_Butter_2007_USA:GA(PB)	Tennessee str. CFSAN001371	USA:GA	peanut butter	JNXX01.0011		454	183850		
1372_Peanut_Butter_2007_USA:GA(PB)	Tennessee str. CFSAN001372	USA:GA	peanut butter	JNXX01.0011		454	183848		SRR1048334
1373_Peanut_Butter_2007_USA:GA(PB)	Tennessee str. CFSAN001373	USA:GA	peanut butter	JNXX01.0011		454	183851		SRR1048312
1374_Clinical_2006_USA:MA(PB)	Tennessee str. CFSAN001374	USA:MA	stool sample	JNXX01.0011	JNXA26.0001	454	183847		SRR1048255
1375_Clinical_2006_USA:MA(PB)	Tennessee str. CFSAN001375	USA:MA	urine sample	JNXX01.0011	JNXA26.0001	454	183850		SRR1048626
1376_Clinical_2004_USA:TN(PB)	Tennessee str. CFSAN001376	USA:TN	human	JNXX01.0011		454	183848		SRR1048242
1377_Peanut_Butter_From_Patient_2007_USA:TN(PB)	Tennessee str. CFSAN001377	USA:TN	peanut butter from sick patient	JNXX01.0011	JNXA26.0001	454	183851		SRR1048238
1378_Peanut_Butter_From_Patient_2007_USA:KY(PB)	Tennessee str. CFSAN001378	USA:KY	peanut butter from sick patient	JNXX01.0011	JNXA26.0001	454	183847		SRR1048247
1379_Peanut_Butter_2009_USA:GA(PB)	Tennessee str. CFSAN001379	USA:GA	peanut butter	JNXX01.0011	JNXA26.0001	454	183850		SRR1048331
1380_Blood_Meal_2010_USA:NY(PB)	Tennessee str. CFSAN001380	USA:NY	blood meal	JNXX01.0011		454	183848		SRR1048311
1381_Hydrolyzed_Vegetable_Protein_Powder_2010_USA:NV(PB)	Tennessee str. CFSAN001381	USA:NV	hydrolyzed vegetable protein powder	JNXX01.0189	JNXA26.0016	454	183851		SRR1048335
1382_Peanut_Butter_2007_USA:GA(PB)	Tennessee str. CFSAN001382	USA:GA	peanut butter	JNXX01.0011		454	183847		SRR1048290
1383_Peanut_Butter_2007_USA:GA(PB)	Tennessee str. CFSAN001383	USA:GA	peanut butter	JNXX01.0011		454	183850		SRR1048315
1384_Peanut_Butter_2007_USA:GA(PB)	Tennessee str. CFSAN001384	USA:GA	peanut butter	JNXX01.0010		454	183848		SRR1048298
1385_Peanut_Butter_2007_USA:GA(PB)	Tennessee str. CFSAN001385	USA:GA	peanut butter	JNXX01.0011		454	183851		SRR1048285
1386_Environmental_2006_USA:GA(PB)	Tennessee str. CFSAN001386	USA:GA	environmental	JNXX01.0011		454	183847		SRR1048622
1387_Peanut_Butter_2007_USA:GA(PB)	Tennessee str. CFSAN001387	USA:GA	peanut butter	JNXX01.0010		454	183850		SRR1048319
1388_Peanut_Butter_2007_USA:GA(PB)	Tennessee str. CFSAN001388	USA:GA	peanut butter	JNXX01.0011		454	183848		SRR1048236
1389_Peanut_Butter_2007_USA:GA(PB)	Tennessee str. CFSAN001389	USA:GA	peanut butter	JNXX01.0011		454	183851		SRR1048305
1390_Peanut_Butter_2007_USA:GA(PB)	Tennessee str. CFSAN001390	USA:GA	peanut butter	JNXX01.0011		454	183847		SRR1048239
2961_Produce_Field_Drag_Swab_2012_USA:NY	Tennessee str. CFSAN002961	USA:NY	produce field—drag swab			Miseq	183850		SRR1012301
3018_Peanut_Butter_2007_USA:NY	Tennessee str. CFSAN003018	USA:NY	peanut butter	JNXX01.0011		Miseq	183850		SRR949423
3019_Peanut_Butter_2007_USA:NY	Tennessee str. CFSAN003019	USA:NY	peanut butter	JNXX01.0011		Miseq	183850		SRR949424
3170_Cilantro_2011_USA:FL	Tennessee str. CFSAN003170	USA:FL	cilantro			Miseq	186035		SRR1033577
3171_Cilantro_2011_USA:FL	Tennessee str. CFSAN003171	USA:FL	cilantro			Miseq	186035		SRR1033550
3172_Cilantro_2011_USA:FL	Tennessee str. CFSAN003172	USA:FL	cilantro			Miseq	186035		SRR952679
3173_Cilantro_2011_USA:FL	Tennessee str. CFSAN003173	USA:FL	cilantro			Miseq	186035		SRR1033559
3174_Cilantro_2011_USA:FL	Tennessee str. CFSAN003174	USA:FL	cilantro			Miseq	186035		SRR1033540
3175_Cilantro_2011_USA:FL	Tennessee str. CFSAN003175	USA:FL	cilantro			Miseq	186035		SRR1033508
3176_Cilantro_2011_USA:FL	Tennessee str. CFSAN003176	USA:FL	cilantro			Miseq	186035		SRR1033543
3186_Cilantro_2011_USA:NY	Tennessee str. CFSAN003186	USA:NY	cilantro			Miseq	186035		SRR1043945
3187_Cilantro_2011_USA:NY	Tennessee str. CFSAN003187	USA:NY	cilantro			Miseq	186035		SRR1041887
3188_Cilantro_2011_USA:NY	Tennessee str. CFSAN003188	USA:NY	cilantro			Miseq	186035		SRR1043946
3189_Cilantro_2011_USA:NY	Tennessee str. CFSAN003189	USA:NY	cilantro			Miseq	186035		SRR1033469
3190_Cilantro_2011_USA:NY	Tennessee str. CFSAN003190	USA:NY	cilantro			Miseq	186035		SRR1036442
3191_Cilantro_2011_USA:NY	Tennessee str. CFSAN003191	USA:NY	cilantro			Miseq	186035		SRR1036432
3192_Cilantro_2011_USA:NY	Tennessee str. CFSAN003192	USA:NY	cilantro			Miseq	186035		SRR1033568
3193_Cilantro_2011_USA:NY	Tennessee str. CFSAN003193	USA:NY	cilantro			Miseq	186035		SRR1041894
3194_Cilantro_2011_USA:NY	Tennessee str. CFSAN003194	USA:NY	cilantro			Miseq	186035		SRR952681
3196_Cilantro_2011_USA:NY	Tennessee str. CFSAN003196	USA:NY	cilantro			Miseq	186035		SRR1033564
3197_Cilantro_2011_USA:NY	Tennessee str. CFSAN003197	USA:NY	cilantro			Miseq	186035		SRR1033473
3198_Cilantro_2011_USA:NY	Tennessee str. CFSAN003198	USA:NY	cilantro			Miseq	186035		SRR1033528
3199_Cilantro_2012_USA:OH	Tennessee str. CFSAN003199	USA:OH	cilantro			Miseq	186035		SRR1033579
3202_Cilantro_2012_USA:OH	Tennessee str. CFSAN003202	USA:OH	cilantro			Miseq	186035		SRR1036448
3203_Cilantro_2012_USA:OH	Tennessee str. CFSAN003203	USA:OH	cilantro			Miseq	186035		SRR1036443
5186_Celery_Stalk_Leaf_2011_USA:IL	Tennessee str. CFSAN005186	USA:IL	celery stalk and leaf	JNXX01.0112		Miseq	186035		SRR1048302
5226_Environmental_Swab_2010_USA:UT	Tennessee str. CFSAN005226	USA:UT	swab		JNXA26.0001	Miseq	186035		SRR1049678
5302_Sunflower_Kernels_2010_China	Tennessee str. CFSAN005302	China	sunflower kernels	JNXX01.0002		Miseq	186035		SRR1049693
1337_*S*.Kentucky	Kentucky str. CFSAN001337	USA:PA	fecal sample			454	66693	AOYZ00000000	SRR955293
1083_*S*.Cubana	Cubana str. CFSAN001083	Philippines	dessicated coconut			Miseq	167394	APAG00000000	SRR955257

Libraries were constructed from cilantro-derived samples using the Nextera XT DNA sample preparation kit (Illumina, San Diego, CA), and whole-genome sequencing was performed on a MiSeq^TM^ benchtop sequencer (Illumina, San Diego, CA), using 500-cycle paired-end reagent kit v2.

### Genome assembly and annotation

De novo assemblies were created for each isolate, using Roche Newbler package (v. 2.6), CLC Genomic Workbench 6.5.1, and SMRT analysis 2.0.1, for isolates sequenced by 454, Miseq^TM^, and PacBio, respectively. All draft genomes were annotated using NCBI’s Prokaryotic Genomes Automatic Annotation Pipeline (PGAAP, [40]). The reference genome used for mapping reads was CFSAN001339, which is comprised of 1 single circular chromosome. Hence, positional information is specific for the reference. (GenBank accession: CP007505).

Phylogenetic trees were constructed using GARLI [41, 42] under the maximum likelihood criterion. The phylogenetic tree in Fig 2 was constructed using GARLI under the GTR + gamma model of nucleotide evolution. Phylogenetic analyses of the data set, including multiple outgroups, were performed on the concatenated SNP matrix described above.

### Phylogenomic analysis

The raw reads of each sample were mapped to the closed reference genome, CFSAN001339, using Novoalign V2.08.02 (http://www.novocraft.com), and the variants were called using SAMtools and stored in a VCF file [43]. A custom Python script was used to read through each VCF file and construct a SNP matrix for further phylogenetic analyses, as follows. First, we estimated the site SNP allele frequencies of the strongest non-reference allele [43] and placed them into a list by collecting all of the instances which met the criteria of being present at positions in the reference where one or more isolates differed with a read depth ≥10 and an allele frequency equal to one. Insertions and deletions (indels) in VCF files were ignored. Second, pileup files were generated for each isolate based on the above-mentioned list to determine the appropriate nucleotide state for positions in the list for each isolate based on the following rules: a) if there was no mapped reads at a position it was treated as missing data; b) if different nucleotides were called at the position, the one with frequency larger than 50% was the consensus call for that position; and c) if different nucleotides were called at a position but none had a frequency larger than 50%, that position for that individual isolate was coded as missing data. Third, the mapped consensus base for each isolate at the reference SNP positions were concatenated in a multiple FASTA file for phylogenetic analysis. The maximum likelihood (ML) tree was constructed using GARLI [41,42] with 200 ML replicates and 1000 bootstrap replicates. All GARLI analyses were performed with the default parameter settings and the GTR+gamma nucleotide substitution model. Detailed descriptions of the data analysis pipeline is available [44] as well as github (see https://github.com/CFSAN-Biostatistics/snp-mutator).

### Accessions

The whole genome shotgun accessions (WGS), Bioproject accession numbers, and metadata for all the isolates sequenced in this study are listed in Table 1. The NCBI accession numbers for the comparative plasmids discussed herein are: *Citrobacter freundii* plasmid pCAV1741-110 (CP011655); *S*. *Typhi* plasmid pHCM2 (AL513384); and *Yersinia pestis* pMT (CP010021).

## Results

### Genome Size, Order and Conservation

We present new draft genomes for 73 *Salmonella* isolates including CFSAN001337, and CFSAN001083, closely related outgroups, *S*. Kentucky and *S*. Cubana, respectively (Table 1). While synteny and genome organization among these isolates was largely conserved, genome size differences were observed due to variations in the presence or absence of several phages and plasmids.

Phylogenomic analysis of the *S*. Tennessee data set, including multiple serovars, was performed on the set of SNPs obtained from the analysis described in the methods. We used the resultant phylogenetic trees to make hypotheses about both the evolution of *S*. Tennessee subtypes and the outbreak strains and also to support traceback investigations.

A list of genes from which the SNPs that characterize the *S*. Tennessee clade were derived is provided in Table 2. A representative SNP from each of these genes is also provided in the table along with the subgroup that it defines the SNP base pair coordinates. Many of these genes were annotated previously with assigned names and functions; however, additional regions that provided signature SNPs are hypothetical and, as such, are cross-referenced by locus tags only. It is notable that a partial and select set of SNPs from these genes are nonsynonymous, and many cluster two or more *S*. Tennessee subgroups together, as shown in Table 2 and Fig 1, and many are protein-altering in nature. These data are intriguing given an NGS report documenting positive selection among a significant subset of core genes in adapted *Salmonella* serovars [45].

**Table 2:** Annotation of clade-specific SNPs found in serovar S. Tennessee.

Location	Accession	Annot.	Locus_tag	Pos. in coding	Nuc. change	Amino acid change	Syn/Non	Strand	Product name
**Clade C1**									
**1361235**	**CP007505**	**coding**	**SEET0819_06395**	**22**	**GAT->TAT**	**D->Y**	**N**	**-**	**transaldolase**
**Node1**									
**1042280**	**CP007505**	**coding**	**SEET0819_04945**	**14**	**TCG->TTG**	**S->L**	**N**	**+**	**phosphate-starvation-inducible protein PsiE**
**1486959**	**CP007505**	**coding**	**SEET0819_06990**	**1203**	**CTG->CTA**	**L->L**	**S**	**+**	**chitinase**
**248590**	**CP007505**	**coding**	**SEET0819_01260**	**2258**	**CAC->CTC**	**H->L**	**N**	**-**	**maltodextrin phosphorylase**
**4310906**	**CP007505**	**coding**	**SEET0819_20670**	**799**	**GAA->TAA**	**E->Stop**	**N**	**-**	**hypothetical protein**
**Clade C2**									
**1261850**	**CP007505**	**coding**	**SEET0819_05950**	**646**	**CGC->AGC**	**R->S**	**N**	**+**	**UDP-N-acetylmuramate:L-alanyl-gamma-D-glutamyl-meso-diaminopimelate ligase**
**1325788**	**CP007505**	**coding**	**SEET0819_06255**	**711**	**CTG->CTA**	**L->L**	**S**	**-**	**multifunctional aminopeptidase A**
**1559936**	**CP007505**	**intergenic**			**C->T**				
**1659427**	**CP007505**	**coding**	**SEET0819_07735**	**609**	**ATG->ATA**	**M->I**	**N**	**+**	**multicopper oxidase**
**2013944**	**CP007505**	**coding**	**SEET0819_09365**	**212**	**CCG->CTG**	**P->L**	**N**	**+**	**hypothetical protein**
**266867**	**CP007505**	**coding**	**SEET0819_01325**	**771**	**CCG->CCA**	**P->P**	**S**	**+**	**ribokinase**
**2745712**	**CP007505**	**coding**	**SEET0819_12935**	**360**	**GCC->GCT**	**A->A**	**S**	**+**	**peptide ABC transporter ATP-binding protein**
**2967521**	**CP007505**	**coding**	**SEET0819_14105**	**1042**	**GCT->ACT**	**A->T**	**N**	**+**	**transcriptional regulator**
**2977833**	**CP007505**	**coding**	**SEET0819_14155**	**400**	**TTC->ATC**	**F->I**	**N**	**-**	**dimethyl sulfoxide reductase**
**3099086**	**CP007505**	**coding**	**SEET0819_14720**	**358**	**GGT->AGT**	**G->S**	**N**	**-**	**XRE family transcriptional regulator**
**4290447**	**CP007505**	**coding**	**SEET0819_20620**	**1930**	**GGC->AGC**	**G->S**	**N**	**+**	**large repetitive protein**
**4415903**	**CP007505**	**intergenic**			**T->G**				
**4675774**	**CP007505**	**coding**	**SEET0819_22430**	**1074**	**TAC->TAT**	**Y->Y**	**S**	**+**	**S-adenosylmethionine synthetase**
**4779893**	**CP007505**	**coding**	**SEET0819_22980**	**627**	**CTG->CTA**	**L->L**	**S**	**+**	**disulfide oxidoreductase**
**4862200**	**CP007505**	**coding**	**SEET0819_23370**	**1544**	**AGC->AAC**	**S->N**	**N**	**-**	**DEAD/DEAH box helicase**
**968881**	**CP007505**	**coding**	**SEET0819_04570**	**2709**	**CTG->CTA**	**L->L**	**S**	**+**	**DNA-directed RNA polymerase subunit beta**
**Clade C3**									
**213029**	**CP007505**	**coding**	**SEET0819_01115**	**2210**	**GAT->GTT**	**D->V**	**N**	**+**	**transcription accessory protein**
**224483**	**CP007505**	**coding**	**SEET0819_01165**	**1533**	**CTG->CTT**	**L->L**	**S**	**-**	**maltose phosphorylase**
**281038**	**CP007505**	**coding**	**SEET0819_01400**	**142**	**TGG->TGT**	**W->C**	**N**	**-**	**leucine/isoleucine/valine transporter permease subunit**
**401342**	**CP007505**	**coding**	**SEET0819_01890**	**3**	**GTG->GTA**	**V->V**	**S**	**-**	**hypothetical protein**
**416161**	**CP007505**	**coding**	**SEET0819_01960**	**57**	**CTG->CTA**	**L->L**	**S**	**+**	**bifunctional glyoxylate/hydroxypyruvate reductase B**
**451802**	**CP007505**	**coding**	**SEET0819_02130**	**196**	**ACG->CCG**	**T->P**	**N**	**+**	**xylulose kinase**
**492375**	**CP007505**	**coding**	**SEET0819_02295**	**648**	**TTC->TTT**	**F->F**	**S**	**-**	**glycosyl transferase**
**516347**	**CP007505**	**intergenic**			**C->A**				
**702891**	**CP007505**	**intergenic**			**A->G**				
**766356**	**CP007505**	**coding**	**SEET0819_03620**	**777**	**ACC->ACA**	**T->T**	**S**	**+**	**phospholipase A**
**972049**	**CP007505**	**coding**	**SEET0819_04585**	**561**	**GGA->GGT**	**G->G**	**S**	**+**	
**1049743**	**CP007505**	**coding**	**SEET0819_04975**	**48**	**CTG->CTA**	**L->L**	**S**	**+**	**maltose-binding protein**
**1065827**	**CP007505**	**coding**	**SEET0819_05055**	**429**	**TTC->TTT**	**F->F**	**S**	**+**	**aromatic amino acid aminotransferase**
**1188083**	**CP007505**	**coding**	**SEET0819_05585**	**428**	**TAT->TGT**	**Y->C**	**N**	**-**	**fumarate reductase**
**1201717**	**CP007505**	**intergenic**			**T->A**				
**1255014**	**CP007505**	**coding**	**SEET0819_05920**	**2699**	**GAG->GGG**	**E->G**	**N**	**+**	**hypothetical protein**
**1271868**	**CP007505**	**coding**	**SEET0819_05995**	**351**	**GGA->GGG**	**G->G**	**S**	**+**	**inosose dehydratase**
**1315853**	**CP007505**	**coding**	**SEET0819_06210**	**94**	**ACC->CCC**	**T->P**	**N**	**+**	**toxin-antitoxin biofilm protein TabA**
**1403676**	**CP007505**	**coding**	**SEET0819_06580**	**1151**	**CCC->CAC**	**P->H**	**N**	**+**	**sigma-54 dependent transcriptional regulator**
**1583841**	**CP007505**	**coding**	**SEET0819_07400**	**275**	**TAT->TTT**	**Y->F**	**N**	**+**	**transcriptional regulator**
**1623141**	**CP007505**	**coding**	**SEET0819_07560**	**2526**	**ATG->ATA**	**M->I**	**N**	**+**	**preprotein translocase subunit SecA**
**2002950**	**CP007505**	**intergenic**			**T->G**				
**2293435**	**CP007505**	**coding**	**SEET0819_10755**	**774**	**GAG->GAA**	**E->E**	**S**	**+**	**LysR family transcriptional regulator**
**2402138**	**CP007505**	**coding**	**SEET0819_11250**	**447**	**GAG->GAC**	**E->D**	**N**	**+**	**peptidase M15**
**2462555**	**CP007505**	**coding**	**SEET0819_11600**	**552**	**CTG->CTT**	**L->L**	**S**	**-**	**hypothetical protein**
**2478416**	**CP007505**	**coding**	**SEET0819_11660**	**1670**	**CCT->CTT**	**P->L**	**N**	**+**	**Clp protease ClpX**
**2488851**	**CP007505**	**coding**	**SEET0819_11710**	**154**	**CTG->TTG**	**L->L**	**S**	**+**	**leucine-responsive transcriptional regulator**
**2547852**	**CP007505**	**coding**	**SEET0819_11930**	**537**	**ATG->ATA**	**M->I**	**N**	**+**	**amino acid:proton symporter**
**2560677**	**CP007505**	**coding**	**SEET0819_11970**	**593**	**GAT->GCT**	**D->A**	**N**	**+**	**paraquat-inducible membrane protein A**
**2592656**	**CP007505**	**coding**	**SEET0819_12155**	**1222**	**GCC->CCC**	**A->P**	**N**	**-**	**Pyoverdin chromophore biosynthetic protein pvcC**
**2693215**	**CP007505**	**coding**	**SEET0819_12675**	**287**	**GTA->GCA**	**V->A**	**N**	**+**	**membrane protein**
**2823636**	**CP007505**	**coding**	**SEET0819_13360**	**692**	**CTC->CAC**	**L->H**	**N**	**+**	**cyclic di-GMP regulator CdgR**
**2901526**	**CP007505**	**coding**	**SEET0819_13765**	**303**	**GTG->GTT**	**V->V**	**S**	**+**	**secretion system apparatus protein SsaU**
**2902776**	**CP007505**	**coding**	**SEET0819_13780**	**1352**	**ATT->AAT**	**I->N**	**N**	**-**	**multidrug transporter**
**2915852**	**CP007505**	**intergenic**			**A->G**				
**2926925**	**CP007505**	**coding**	**SEET0819_13915**	**214**	**TAC->CAC**	**Y->H**	**N**	**-**	**glutathione S-transferase**
**2990295**	**CP007505**	**coding**	**SEET0819_14220**	**633**	**GAC->GAA**	**D->E**	**N**	**-**	**malonic semialdehyde reductase**
**3062233**	**CP007505**	**coding**	**SEET0819_14570**	**559**	**ACG->GCG**	**T->A**	**N**	**+**	**TetR family transcriptional regulator**
**3141458**	**CP007505**	**coding**	**SEET0819_14930**	**1564**	**GTG->TTG**	**V->L**	**N**	**-**	**hypothetical protein**
**3166809**	**CP007505**	**coding**	**SEET0819_15030**	**319**	**TTT->ATT**	**F->I**	**N**	**+**	**hypothetical protein**
**3288278**	**CP007505**	**coding**	**SEET0819_15625**	**248**	**CGC->CAC**	**R->H**	**N**	**-**	**peptide chain release factor 1**
**3353430**	**CP007505**	**coding**	**SEET0819_15955**	**24**	**AAG->AAA**	**K->K**	**S**	**-**	**transcriptional regulator**
**3461711**	**CP007505**	**coding**	**SEET0819_16570**	**42**	**CAG->CAA**	**Q->Q**	**S**	**-**	**glycosyl hydrolase family 88**
**3556791**	**CP007505**	**coding**	**SEET0819_17120**	**110**	**ACT->AAT**	**T->N**	**N**	**-**	**acyl carrier protein**
**3564805**	**CP007505**	**intergenic**			**T->C**				
**3631271**	**CP007505**	**coding**	**SEET0819_17515**	**656**	**ACC->ATC**	**T->I**	**N**	**+**	**imidazoleglycerol-phosphate dehydratase**
**3835332**	**CP007505**	**coding**	**SEET0819_18455**	**244**	**TCT->GCT**	**S->A**	**N**	**+**	**transcriptional regulator**
**3976686**	**CP007505**	**intergenic**			**T->C**				
**3977014**	**CP007505**	**coding**	**SEET0819_19075**	**177**	**TCG->TCT**	**S->S**	**S**	**+**	**integrase**
**3981933**	**CP007505**	**coding**	**SEET0819_19095**	**732**	**GCT->GCA**	**A->A**	**S**	**-**	**hypothetical protein**
**3981936**	**CP007505**	**coding**	**SEET0819_19095**	**729**	**CCG->CCA**	**P->P**	**S**	**-**	**hypothetical protein**
**3981954**	**CP007505**	**coding**	**SEET0819_19095**	**711**	**ACG->ACT**	**T->T**	**S**	**-**	**hypothetical protein**
**3981966**	**CP007505**	**coding**	**SEET0819_19095**	**699**	**GCC->GCA**	**A->A**	**S**	**-**	**hypothetical protein**
**3981968**	**CP007505**	**coding**	**SEET0819_19095**	**697**	**GCC->TCC**	**A->S**	**N**	**-**	**hypothetical protein**
**3998142**	**CP007505**	**coding**	**SEET0819_19180**	**105**	**GGG->GGA**	**G->G**	**S**	**-**	**hypothetical protein**
**4007005**	**CP007505**	**intergenic**			**T->G**				
**4007065**	**CP007505**	**intergenic**			**A->C**				
**4007316**	**CP007505**	**intergenic**			**C->T**				
**4007326**	**CP007505**	**intergenic**			**A->G**				
**4007341**	**CP007505**	**intergenic**			**G->A**				
**4007389**	**CP007505**	**intergenic**			**A->G**				
**4007514**	**CP007505**	**intergenic**			**G->A**				
**4007522**	**CP007505**	**intergenic**			**T->C**				
**4007523**	**CP007505**	**intergenic**			**A->C**				
**4007528**	**CP007505**	**intergenic**			**A->G**				
**4007551**	**CP007505**	**intergenic**			**A->G**				
**4007874**	**CP007505**	**coding**	**SEET0819_19270**	**528**	**GAT->GAA**	**D->E**	**N**	**-**	**replication protein**
**4008036**	**CP007505**	**coding**	**SEET0819_19270**	**366**	**TCC->TCA**	**S->S**	**S**	**-**	**replication protein**
**4008048**	**CP007505**	**coding**	**SEET0819_19270**	**354**	**AAC->AAT**	**N->N**	**S**	**-**	**replication protein**
**4008441**	**CP007505**	**intergenic**			**G->A**				
**4008502**	**CP007505**	**intergenic**			**A->G**				
**4008537**	**CP007505**	**intergenic**			**T->G**				
**4010438**	**CP007505**	**coding**	**SEET0819_19300**	**124**	**GGC->TGC**	**G->C**	**N**	**+**	**hypothetical protein**
**4010464**	**CP007505**	**coding**	**SEET0819_19300**	**150**	**CAT->CAG**	**H->Q**	**N**	**+**	**hypothetical protein**
**4010470**	**CP007505**	**coding**	**SEET0819_19300**	**156**	**GTC->GTT**	**V->V**	**S**	**+**	**hypothetical protein**
**4010508**	**CP007505**	**coding**	**SEET0819_19300**	**194**	**TCG->TTG**	**S->L**	**N**	**+**	**hypothetical protein**
**4010580**	**CP007505**	**coding**	**SEET0819_19300**	**266**	**GCT->GTT**	**A->V**	**N**	**+**	**hypothetical protein**
**4010593**	**CP007505**	**coding**	**SEET0819_19300**	**279**	**GGA->GGG**	**G->G**	**S**	**+**	**hypothetical protein**
**4012278**	**CP007505**	**coding**	**SEET0819_19335**	**45**	**TTC->TTT**	**F->F**	**S**	**+**	**regulatory protein**
**4012281**	**CP007505**	**coding**	**SEET0819_19335**	**48**	**TAC->TAT**	**Y->Y**	**S**	**+**	**regulatory protein**
**4324886**	**CP007505**	**intergenic**			**G->A**				
**4669017**	**CP007505**	**intergenic**			**T->A**				
**Clade C4**									
**1068806**	**CP007505**	**coding**	**SEET0819_05080**	**360**	**AAT->AAA**	**N->K**	**N**	**-**	**lipoprotein**
**1935930**	**CP007505**	**coding**	**SEET0819_08985**	**966**	**AAT->AAC**	**N->N**	**S**	**+**	**S-adenosylmethionine:tRNA ribosyltransferase-isomerase**
**3265625**	**CP007505**	**coding**	**SEET0819_15515**	**211**	**GGG->AGG**	**G->R**	**N**	**+**	**hypothetical protein**
**Node2**									
**1001360**	**CP007505**	**coding**	**SEET0819_04720**	**856**	**GCG->TCG**	**A->S**	**N**	**+**	**isocitrate dehydrogenase**
**1086809**	**CP007505**	**coding**	**SEET0819_05115**	**8047**	**GAT ->AAT**	**D->N**	**N**	**+**	**membrane protein**
**1389803**	**CP007505**	**coding**	**SEET0819_06540**	**1764**	**ATT->ATC**	**I->I**	**S**	**-**	**type I restriction-modification protein subunit S**
**2186959**	**CP007505**	**coding**	**SEET0819_10205**	**29**	**TCT->TTT**	**S->F**	**N**	**-**	**LPS biosynthesis protein**
**314299**	**CP007505**	**coding**	**SEET0819_01560**	**115**	**GTC->ATC**	**V->I**	**N**	**+**	**copper resistance protein**
**331099**	**CP007505**	**intergenic**			**G->A**				
**334506**	**CP007505**	**intergenic**			**A->T**				
**4620448**	**CP007505**	**intergenic**			**C->T**				

### Genetic Variation within the Tennessee serovar

As shown in Table 1, the isolates derived from the peanut butter-derived sources, including samples obtained from outbreak-associated foods and clinical samples, were observed to have distinct PFGE profiles. The *S*. Tennessee isolates from the 2006–2007 outbreak displayed four closely related primary (XbaI-derived) PFGE patterns: JNXX01.0010, JNXX01.0011, JNXX01.0026. [2,6,10]. Secondary patterns (BlnI-derived) for PFGE type JNXX01.0011 were all classified as JNXA26.0001.

A set of non-peanut butter-derived *S*. Tennessee isolates also exhibited one of the same PFGE patterns as found in the peanut butter-derived samples: JNXX01.0011. Samples in this set included isolates from fishmeal (CFSAN001339 and CFSAN001342); lamb from New Zealand (CFSAN001340), poultry (CFSAN001341), cotton seeds (CFSAN001343), and soy beans (CFSAN001344).

Other *S*. Tennessee serovar isolates included in this study that came from non-peanut butter sources also exhibited different PFGE patterns; for example: celery (CFSAN005186, PFGE pattern JNXX01.0112); an environmental swab, (CFSAN005226, PFGE pattern JNXA26.0001); sunflower kernels from China (CFSAN005302, PFGE pattern JNXX01.0002); hydrolyzed vegetable protein powder (CFSAN001381, primary PFGE pattern JNXX01.0189, a secondary PFGE pattern of JNXA26.0016; this isolate also carried a 30kb phage PsP3, discussed further below).

The PFGE patterns for all 22 *S*. Tennessee isolates obtained from cilantro were identical (JNXX01.0011). Analyses of these whole genome sequences revealed that all 22 cilantro isolates of *S*. Tennessee formed a distinct group. Our PFGE and WGS analyses suggest a common source for these isolates, even though the isolates were collected from 3 states. Cilantro is typically grown in only 2 or 3 areas of the country and provided to the consumer through a complex distribution network, such that the state of collection for this study may not be the state where the cilantro was grown. Further examination of this distribution network revealed that eight of these isolates originated in California, and five originated in Mexico; the origin of the remaining nine could not be determined.

A recent report on the potential enhanced virulence of the peanut butter-derived *Salmonella* isolates [46] led us to compare the genic origin of the SNPs found within the peanut butter-derived strains in our study to the SNPs found in isolates obtained from non-peanut butter sources (Table 2). Many of the observed SNP differences were non-synonymous, coding for amino-acid changes. Further investigation is needed to determine whether or not these coding changes result in virulence changes.

Cluster analyses also revealed 13 isolates with the same PFGE pattern as the most common pattern in this outbreak (JNXX01.0011) that do not belong in the outbreak clade. These isolates include those collected from the 2008 peanut butter outbreak, three clinical isolates from MA, two clinical isolate from IA, and seven isolates from animal feed. Additionally, eight of the 13 clinical and two of the environmental isolates in this study are in the outbreak clade. None of the SNPs we identified in this study were specific to clinical or environmental sources. It is noteworthy that no increases in substitutions were identified among the isolates that passed through patients compared to their environmental sources. Had there been an increase or expansion in genetic diversity among the clinical isolates we studied in comparison to isolates collected from other food and environmental sources, we would have expected that genetic diversity to have been visible as longer branch lengths among the terminal tree nodes leading back to the clinical isolates found in the tree.

### Phylogenetic analysis

The phylogenetic tree arising from this analysis is depicted in Fig 2. For discussion purposes, we have identified four intra-serovar clades, C1-C4. C1 consists of 31 isolates, all closely related, containing both clinical and environmental sources, and each separated by a single SNP. The node (node 1) defining this clade consists of four unique SNPs. C2 is a small clade of three isolates defined by 16 SNPs. C3 contains 22 isolates, differentiated by 82 total SNPs. All of the C3 isolates were obtained from a cilantro food source. Node 2, defining clades C2-C4, contains 8 unique SNPs. The Tennessee clade is identified by a total of 1,153 SNPs, most of which (1,061) map to the long branch separating the outgroups from the Tennessee-specific isolates. Interestingly, the singleton-containing branches consisting of isolate numbers 1381, 2961, and 5226 all contain large mobile elements.

### Specific Genes and SNP-based genetic variation defining the Tennessee serovar

A total of 114 SNPs were found in S. Tennessee genes, and including representatives from each of the four S. Tennessee clades (Table 2). Although many of these changes are synonymous, many others are non-synonymous (discussed further below). Similar to earlier studies, we observed changes in the *S*. *enterica* multicopper oxidase gene, (locus tag SEET0819_07735, position 609), a gene reported to harbor many changes within *S*. Enteritidis strains. Although the gene and protein alignments show many of the same non-synonymous SNP differences that appear in all the *S*. Tennessee isolates we examined [21], we also identified a change in the S. Tennessee serovars at genome position 1659427, resulting in a M-I amino acid change in the multicopper oxidase gene.

Other non-synonymous SNP changes affected genes involved in redox-type chemical reactions. In particular, we found an F-I change in the dimethyl sulfoxide reductase gene at position 2977833; this is a molybdenum-containing enzyme capable of reducing dimethyl sulfoxide (DMSO) to dimethyl sulfide (DMS). This enzyme serves as the terminal reductase under anaerobic conditions in some bacterial species, with DMSO serving as the terminal electron acceptor. At genome position 1261850 there was a change from R-S within the UDP-N-acetylmuramate: L-alanyl-gamma-D-glutamyl-meso-diaminopimelate ligase gene, a gene involved in peptidoglycan recycling that reutilizes the intact tripeptide L-alanyl-gamma-D-glutamyl-meso-diaminopimelate by linking it to UDP-N-acetylmuramic acid. At position 136125 we found a change from D-Y in the transaldolase gene, an enzyme of the non-oxidative phase of the pentose phosphate pathway.

Eleven non-synonymous SNPs fell within hypothetical proteins (at positions 4310906, 2013944, 3265625, 1255014, 3141458, 3166809, 3981968, 4010438, 4010464, 4010508 and 4010580). One SNP mapped to a lipoprotein (1068806), while another fell within a large repetitive protein (4290447).

Many of SNPs resulting in amino acid changes were involved in transcriptional regulation or DNA structural modifications related to gene expression. These include changes in the XRE family of transcriptional regulator genes at position 3099086; three generic transcriptional regulator changes at positions 2967521, 1583841, and 3835332, and a change at position 4862200, a S-N alteration in the DEAD/DEAH box helicase gene, a family of DNA-unwinding and RNA-processing proteins (Table 2).

### Mobile Elements

Natural selection has been reported in *Salmonella* and appears to be a major component of the evolution of this pathogen [33, 47]. Some of the variable genes in *Salmonella* are found in the mobilome, consisting of phages and plasmids, which are often the most promiscuous portions of the bacterial genomes [31, 30, 48–50]. This evolutionary strategy could provide a mechanism whereby highly selected genes could be shaped by natural selection, and then be easily distributed among the members of a serotype and other, more distant, lineages through mobile genetic elements.

We have also identified several new plasmids (Table 3) suggesting that whole genome sequencing will continue to provide novel information about the *Salmonella* genome. Genes contributing to virulence are often carried on mobile elements, therefore it is especially important to study these elements in pathogenic strains.

**Table 3:** Mobile elements and plasmids found in the S. Tennessee serovar.

	12kb_Insertion	30kb_PhagePsP3	63kb_Insertion	110kb_PhageSSU5	260kb_PlasmidR478
CFSAN002961		+			+
CFSAN001365				+	
CFSAN001368				+	
CFSAN001387				+	
CFSAN001381		+			
CFSAN005226	+	+	+		

Notes: The 63kb_Insertion region was found only in CFSAN005226, which is a singleton in the tree.

CFSAN002961 also has a very large plasmid (similar to Serratia marcescens plasmid R478, 274762 bp) that is not shared by any other isolates. CFSAN002961 is also a singleton in the tree.

CFSAN001365 (2004 MA clinical), 1368 (2007 GA peanut butter), and 1387 (2007 GA peanut butter) are isolates from clade C1, and they seem to share a 110 kb phage (see text for further discussion), which is found to be similar to Salmonella phage SSU5 (103299 bp).

We found five mobile elements within the Tennessee serovar (Table 3). CFSAN001365 (2004 MA clinical), CFSAN001368 (2007 GA peanut butter), and CFSAN001387 (2007 GA peanut butter) cluster together in clade C1, and they all share a 110 kb phage, which is found to be similar to *Salmonella* phage SSU5 (103,299 bp). This phage was originally described in *S*. *enterica* serovar Typhimurium, and its whole genome was sequenced and analyzed [51]. The double-stranded DNA genome of SSU5 encodes 130 open reading frames with one tRNA for asparagine. Genomic analysis revealed that SSU5 might be the phylogenetic origin of cryptic plasmid pHCM2, harbored by Salmonella Typhi CT18. Our investigation shows that this sequence shares 77% sequence similarity (query cover) with approximately 99% sequence identity with the *Citrobacter freundii* plasmid pCAV1741-110 and with *S*. Typhi plasmid pHCM2. Further, it shows some similarity (57%) with 90% sequence identity with the virulence-associated plasmid pMT from *Yersinia pestis*. Further investigation is warranted to determine whether or not this sequence is carried on a distinct plasmid in Salmonella.

Table 3 lists the remaining mobile elements identified here in *S*. Tennessee, including a 12 kb insertion, a 30 kb PhagePsP3-like element, a 63 kb insertion, the previously mentioned 110 kb phage (SSU5-like), and a 260 kb plasmid R478-like mobile element [52]. Comparison of Figs 1 and 2 shows the relationship between the mobile elements and the phylogenetic signal which accompanies each.

## Discussion

The phylogenomic analysis of the *S*. Tennessee serovar samples contained in this study demonstrates a number of important points that are relevant to foodborne outbreak investigations. First, these results continue to underscore the power of whole genome sequencing in outbreak investigations. Although in most cases PFGE patterns will provide sufficient resolution to determine the relationships between closely related isolates, in some cases additional resolution provides information that would not be available from PFGE patterns alone. Second, the power of genome sequencing leads to the identification of classes of SNPs and mobile elements that help us understand the molecular mechanisms of pathogen virulence. This knowledge will serve to establish new typing methods that are focused on particular genetic changes present in genomes, and may also lead to insights that will affect the development of treatments designed to protect human health.

Like other molecular epidemiology studies of *Salmonella* employing genomic technologies [30–34], this work demonstrates that comparative WGS methods can be employed to clearly augment food contamination investigations by genetically linking the implicated sources of contamination with environmental and clinical isolates. The genomic evidence herein corroborates epidemiological conclusions from outbreak investigations based on statistical analysis and source tracking leads. However, with WGS, one can gain additional detailed micro-evolutionary knowledge within the associated outbreak and reference isolates; thus providing additional evidence linking implicated sources to some of the clinical isolates but not to others that might have initially been associated with this foodborne contamination. Moreover, the level of genetic resolution obtained using WGS methods permits delimiting the scope of an outbreak in the context of an investigation, even for the most genetically homogeneous salmonellae [30]. Phylogenetic evolutionary hypotheses can help us identify reliable diagnostic nucleotide motifs (SNPs, rearrangements, and gene presences) for detecting outbreak strains and understanding the mechanisms that drive the outbreak occurrences. These methods allow both the rapid characterization of the genomes of foodborne pathogenic bacteria and can help to identify the particular source of contamination in the food supply.

Using the comparative WGS results and full genomic data reported here we can confirm that some clinical isolates collected during the time of the peanut butter contamination event have the same PFGE Pattern, JNXX01.0011, which has been linked to the implicated environmental isolates previously studied. Importantly, while most of the isolates collected during this time period that share a common PFGE pattern fall into the same clades (Fig 2) with the environmental isolates, several strains known to be unrelated to the outbreak, including historical isolates from earlier analyses, interrupt these lineages, indicating additional potential sources of contamination.

Our results corroborate those from a previous study [30]. We found no apparent increase in substitutions among the clinical isolates that passed through patients compared to the environmental clones of those isolates. Fig 2 shows that both clinical and environmental peanut butter isolates cluster within the same clade, with no apparent differences attributable to human gastrointestinal passage.

From the data presented, as well as from other published data on mobile elements, it would appear that the elements identified herein are not restricted to closely related isolates in the phylogenetic context. For example, a recently discovered *Salmonella* plasmid (pSEEE1729_15) has a DNA sequence similar to an *E*. *coli* 0157:H7 strain EC4115 [53], suggesting that parts of the mobilome may be transferred between enterobacterial species, while raising the possibility of new acquisitions into the *S*. Enteritidis pan genome [48]. Consistent with other studies, we did not find any distinctive differences between isolates recovered from food sources and those obtained from clinical samples. A further comparative analysis of the structure and gene organization in the mobile elements in the isolates recovered from peanut butter will be the subject of a subsequent paper.

Mining the data of these novel *S*. Tennessee genomes should provide new genetic targets for pathogen detection by public health laboratories, and support investigations of outbreaks that consist of closely related *Salmonella* pathogens. Akin to earlier findings of NGS-based differentiation of *S*. Montevideo isolates associated with pepper and spiced meats [30–32], the signature genetic differences uncovered here will provide additional insight into what will likely remain a common pattern of S. Tennessee associated with the food supply. By identifying unique genetic patterns that can rapidly distinguish among multiple serotypes of closely related pathogens and PFGE types, WGS has become an invaluable tool for future molecular epidemiology investigations.

## Conclusions

It appears that, at least in the case of *Salmonella*, the natural variation observed among strains is both stable and sufficient to allow for high-resolution traceback of food and clinical isolates using NGS. It will be interesting to see whether ample genomic diversity can drive similar outcomes in other problematic taxa and closely related *Salmonella* serotypes. By providing the phylogenetic context on which to interpret other facile subtyping approaches that focus on more rapidly evolving genetic markers such as MLVA, rep-PCR, and CRISPRs [6–10, 22] NGS can provide a novel suite of SNPs that will be critical to partitioning common *Salmonella* outbreak strains. Combined with phylogenetic analysis, WGS can illuminate the genetic and evolutionary diversity of important serovars of *Salmonella* and expand our understanding of the associated epidemiological pathways surrounding specific outbreak strains [28, 29, 31, 32].

## Supporting Information

S1 File CFSAN, strain names, assemblies, and WGS accession numbers. (XLSX)
